# Editorial: Early diagnosis in head and neck cancer: advances, techniques, and challenges

**DOI:** 10.3389/froh.2025.1650353

**Published:** 2025-07-09

**Authors:** Natalia Koerich Laureano, Elena Riet Correa Rivero, Giuseppe Pannone, Fernanda Visioli

**Affiliations:** ^1^ Oral Pathology Department, School of Dentistry, Federal University of Rio Grande do Sul, Porto Alegre, Brazil; ^2^ Pathology Department, Federal University of Santa Catarina, Florianópolis, Brazil; ^3^ Department of Clinical and Experimental Medicine, Anatomic Pathology Unit, University of Foggia, Foggia, Italy

**Keywords:** HNSCC (head and neck squamous cell carcinoma), thyroid cancer, ultrasound, radiomic, nomogram, deep learning, epigenetics

**Editorial on the Research Topic**
Early diagnosis in head and neck cancer: advances, techniques, and challenges

Head and neck cancers (HNC) are the 6th most common type of cancer, with an annual incidence estimated to be over 1 million new cases in 2025, an increase of 6% in 3 years. Thyroid cancers (TC) are the 8th most common, with 466,118 new cases in 2022 and an estimated 481,541 new cases by 2025. China alone represents more than half of the global incidence of thyroid cancer, which has increased by 5% in the last 3 years (1).

Despite the advances in therapy, the prognostic values of those cancers have not improved over the last decades, remaining a challenge for clinicians and researchers. This Research Topic addresses original research that explores early diagnosis in HNC and TC.

The advancement in technology has made it possible to identify genetic and epigenetic changes in HNC at an early stage and improve patient prognoses. In order to detect head and neck squamous cell carcinoma (HNSCC) recurrence early, Dal Secco et al. proposed an analytical pipeline to detect somatic variants in saliva liquid biopsy through DNA next-generation sequencing. The study collected saliva samples from 17 non-metastatic HNSCC patients before surgical resection during the 4-year follow-up period. The cDNA evaluation was able to detect changes in low-frequency somatic mutation within 4 months before clinical recurrence in two of three patients who experienced loco-regional relapse. This pilot study brings a promising non-invasive technique to monitoring patient follow-up.

Detection of biomarkers from different samples is important for monitoring cancer development and progression. Zhang et al. elucidate the role of HNRNPC, the most common RNA-binding protein with demethyltransferase activity, which so far is not understood in HNSCC. High levels of HNRNPC were related to poor prognosis in HNSCC patients. HNRNPC knockdown in HNSCC cell lines leads to proliferation, invasion, and malignant transformation abilities. RNA and methylates RNA sequencing analysis identified HNRNPC in cell differentiation, migration, and apoptosis. A mouse xenograft model showed the role of HNRNPC in promoting tumorigenesis and progression. Thus, the authors suggest HNRNPC as a novel predictor of tumor progression and a predictor of HNSCC patient prognoses.

The development of artificial intelligence tools to aid clinical, image, and histopathological diagnosis is becoming a reality in cancer research. However, the previously developed methods still lack good accuracy for clinical application. Zhang et al. proposed a radiomics and deep learning (DL) fusion model based on MRI to distinguish sinonasal squamous cell carcinoma (SNSCC) and sinonasal lymphoma (SNL), two malignant neoplasms with similar manifestations and imaging characteristics. The combined model showed higher accuracy in distinguishing SNSCC and SNL, presenting itself as a non-invasive tool capable of predicting the diagnosis and helping to determine a personalized treatment plan.

Despite increased access to imaging tests such as ultrasound (US) and computed tomography (CT), some rare cancers still face misdiagnosis. Yu et al. presented a rare case of thyroid squamous cell carcinoma that clinically presented as a painful neck mass, diagnosed prior as a cystic solid mass after US, CT, and laryngoscopic screening, and was treated as subacute thyroiditis. Therefore, the diagnosis of thyroid nodules can be challenging.

The Thyroid Imaging, Reporting and Data System (TIRADS) is an ultrasound-based risk stratification system to aid in differentiating between benign and malignant thyroid nodules. However, some stratifications still lack precision in diagnosing malignancy, with a high rate of variation (2). The development of supplementary technologies to enhance the accuracy of thyroid nodule image diagnosis might help clinical decision-making and reduce misdiagnosis. Xie et al. investigated the efficacy of Contrast-Enhanced Ultrasound (CEUS), which assesses the microvascular distribution of lesions and parenchymal perfusion, qualitative and quantitative parameters through multifactor analysis for differentiation of small solid C-TIRADS 4 thyroid nodules. The logistic model demonstrated significantly higher diagnostic performance compared to individual parameters, with an accuracy of 90%. The combination of C-TIRADS with qualitative and quantitative CEUS parameters increases the diagnostic accuracy of malignant thyroid nodules.

To improve the diagnosis of thyroid adenomatoid nodules by ultrasound (TANU), Cheng et al. developed a nomogram that incorporates US-based radiomic features and clinical information. A radiomic nomogram, a clinical nomogram, and a radiomic-clinical nomogram were constructed using machine learning algorithms. The nomograms that incorporated clinical data and radiomic features showed higher accuracy in diagnosing TANU and guiding therapeutic decisions.

Papillary thyroid carcinoma (PTC), the most common TC subtype, generally has a favorable prognosis; however, up to 40% of patients may develop lymph node metastasis (LNM). The LNM screening is performed by ultrasound, although its anatomical characteristics pose challenges in terms of the diagnostic sensitivity of ultrasound. Thus, Hao et al. proposed a clinical prediction model that integrates ultrasound features, clinical parameters, and biochemical markers to provide a practical tool to identify patients at risk of LNM and guide surgical treatment strategies. Regression models identified age, male sex, nodule size, multifocal lesions, capsular contact or invasion, and ill-defined margins as risk variables. A nomogram was created based on those risk factors. Each risk factor produced a cumulative total, which corresponds to a specific value on the risk axis, indicating the likelihood of LNM in patients with PTC. A high score indicated an increased risk of LNM for patients with PTC. The prediction model showed excellent accuracy and can guide clinical decision-making.

The present Research Topic highlights novel approaches in the early diagnosis of HNC and non-invasive techniques with promising results for future clinical application. The prospect of future applicability of noninvasive techniques, such as liquid saliva biopsy, is an important step towards early diagnosis and potentially reducing the high local recurrence rate—currently around 50% within 2 years—which remains a major obstacle to improving patient outcomes. Furthermore, when combined with next-generation sequencing, this enables the identification of new biomarkers to monitor tumor progression and screen patients with a poorer prognosis.

The use of imaging tests is essential for the diagnosis of cancers in anatomical locations that preclude direct visual inspection during clinical care. The use of different imaging diagnostics, enhanced by the use of DL and radiomics, has facilitated the identification of parameters previously imperceptible in human evaluation. The proposed imaging prediction models integrate multiple approaches, underscoring the importance of combining diverse evaluation methods and artificial intelligence to enhance diagnostic accuracy and clinical applicability. Their applicability can reduce incorrect diagnoses and unnecessary additional examinations. Moreover, the combination of these findings with clinical information suggests that clinical prediction models are a possible tool for diagnosis based on information from imaging exams and for screening patients at risk of developing metastases.
